# Finite element analysis of dual small plate fixation and single plate fixation for treatment of midshaft clavicle fractures

**DOI:** 10.1186/s13018-020-01666-x

**Published:** 2020-04-15

**Authors:** Fangxue Zhang, Fancheng Chen, Yuhan Qi, Zhi Qian, Shuo Ni, Zeyuan Zhong, Xu Zhang, Dejian Li, Baoqing Yu

**Affiliations:** 1 Department of Orthopedics, Shanghai Pudong Hospital, Fudan University Pudong Medical Center, No.2800 Gongwei Road, Huinan Town, Pudong New Area, Shanghai City, China; 2 Shanghai Medical College, Fudan University, Shanghai, China; 3 Department of Plastic Surgery, Union Hospital, Tongji Medical College, Huazhong University of Science and Technology, Wuhan, People’s Republic of China

**Keywords:** Finite element analysis, Biomechanics, Clavicle fracture, Dual small plating, Larger single plate constructs

## Abstract

**Background:**

Midshaft clavicle fractures are one of the most familiar fractures. And, dual small plate fixation has been reported as can minimize hardware-related complications. However, the biomechanical properties of the dual small plate fixation have not yet been thoroughly evaluated. Here, we report the results of a finite element analysis of the biomechanical properties of midshaft clavicle fractures treated with dual small plating and superior and anteroinferior single plate fixation.

**Methods:**

A three-dimensional (3D) finite element model of the midshaft clavicle fractures was created, whose 4-mm transverse fracture gap, having an angle < 30 degree and devoid of overlapping triangles, was simulated between the fractured segments of the middle-shaft of the clavicle. The equivalent von Mises stress and displacement of the model was used as the output measures for analysis.

**Results:**

No significant differences were found between dual plating, superior or anteroinferior single plating in cantilever bending, axial compression, and axial torsion. Dual plating with a smaller plate-screw construct is biomechanically eligible to compare with superior and anteroinferior single plate fixation using larger plate-screw constructs.

**Conclusions:**

This study demonstrated that larger plate-screw constructs for the treatment of simple are placed clavicular fractures; however, weight-bearing and exorbitant shoulder activity should be avoided after the operation. Therefore, dual plating may provide a viable option for fixing midshaft clavicle fractures and, thus, may be preferred for patients who need early activity.

## Introduction

Clavicle fractures are among athletes, young individuals, and mainly result from sports injuries, falls, or traffic accidents. And over 80% of clavicle fractures involve the midshaft, and over half of these fractures are displaced in the reason of the relatively narrow cross-section of the bone experiencing excessive torsional or bending stress [1, 2]. Due to high-quality randomized controlled studies reporting, the treatment have changed a lot in the past few decades, significantly decreased rates of nonunion and symptomatic malunion in surgery compared with non-operative treatment [1, 3–8]. For athletes, high return rate, faster return to play, and excellent patient-reported outcomes have been reported after fixation. Consequentially, open reduction internal fixation of midshaft clavicle fractures has become a common treatment approach [1, 3, 4, 6, 9].

Although various fixation methods of midshaft clavicle fractures have been reported by multifarious techniques, plate fixation remains the most established method. Fixations including anterior plate, superior plate, or spiral plate, and, more recently, dual small plate fixation have been reported as can minimize hardware-related complications [2, 7, 9–12]. Plate hardware irritation and prominence are commonly reported as reasons for revision surgeries [3, 6, 13]. On the contrary, higher patient cosmetic acceptability has been reported with small single plate fixation compared with larger, more prominent plates (95% vs 50%, respectively). Recently, excellent clinical outcomes, 100% union rate, and 0% reoperation rate have been reported with dual small plate fixation [7, 10, 11, 13–16]. This has caused clinical interest in dual small plating, to minimize hardware irritation and reoperation rates.

Previous studies have shown good clinical and functional outcomes with dual small orthogonal plating in midshaft clavicular fractures. However, limited biomechanical data exist comparing dual small plating stability under physiological conditions, particularly in comparison with single-plate fixation. As an accurate and effective computational means, finite element analysis (FEA) has received extensive acceptance in the field of orthopedic research [17]. The deeper insight into the stability and functionality of bone constructs can be furnished by the biomechanical studies which use the computational simulation [17–19]. Therefore, the purpose of this study was to compare the biomechanics and evaluate implant stresses and micromotions of 2 methods of plate fixation in midshaft clavicular fractures by using FEA. The conclusions provide a biomechanically based framework in which to consider the application of one or the other approach.

## Material and methods

### Finite element modeling

The computed tomography (CT) scan of the clavicle was acquired from a male volunteer (age 48 years; weight 60 kg; and height 171 cm). Slice thickness of CT images was 0.75 mm (512 × 512 pixels per image). And the geometry of the clavicle model was reconstructed in three-dimensional (3D) geometry format by the software Mimics 15.0 (Materialize Company, Leuven, Belgium) based on the initial 1-mm cuts CT data imported. The volunteer’s medical history excluded comorbidities such as osteoporosis, osteoarthritis, and fractures and cancer. The cortical shell and the inner spongious bone of the clavicle were created based on the Hounsfield values of the bone. The performance of further polishing and the establishment of fracture line were done by the Geomagic Studio Software (3D system Inc., Rock Hill, SC, USA). A 4-mm transverse fracture gap, having an angle < 30 degree and devoid of overlapping triangles, was simulated between the fractured segments of the middle-shaft of the clavicle by the Geomagic Studio Software (3D system Inc, Rock Hill, SC, USA). The 3D models of intramedullary nails, plate, and screws were drawn by the software Creo 3.0 (Parametric Technology Corporation, USA) according to the manufacturer’s pacifications.

**Fig. 1:**
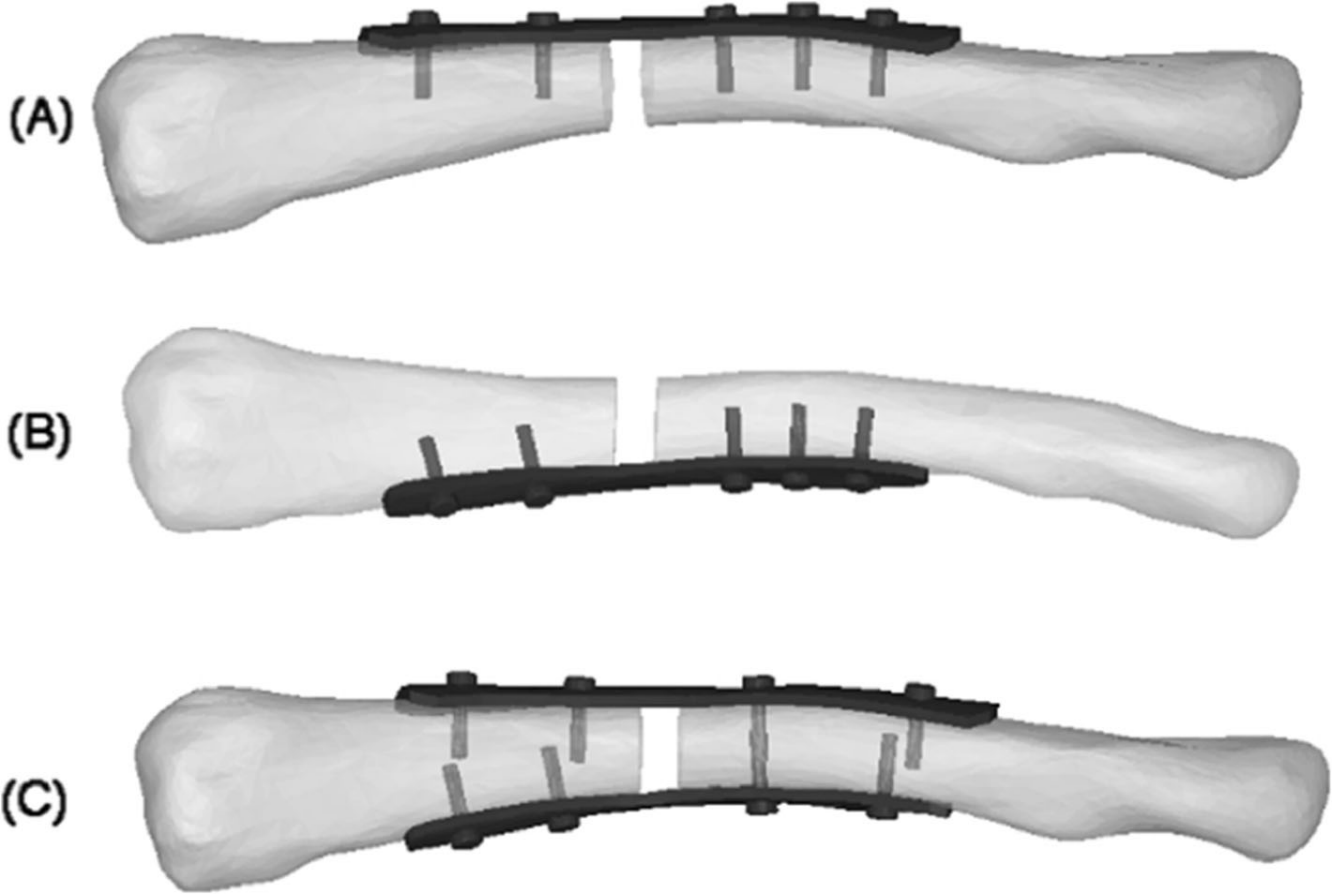
Finite element model of mid-shaft clavicle fractures fixed by the superior plate (**a**), anteroinferior plate (**b**), and dual plate (**c**)

Three types of fixation/implants were modeled and simulated: (1) superior plating with a 6-hole, precontoured, large bend titanium clavicle plate (98-mm length, 11-mm width, 3.3-mm thickness) with a total of five 3.5-mm cortex screws placed bicortically; (2) anteroinferior plating with a 6-hole, precontoured, titanium clavicle plate (95-mm length, 11-mm width, 3.3-mm thickness); and with a total of five 3.5-mm cortex screws placed bicortically; (3) dual small plating with two 6-hole titanium plates placed orthogonally (85-mm length, 11-mm width, 1.6-mm thickness) with a total of four 2.7-mm cortex screws placed bicortically were used for each plate (8 screws total). The dual plating fixation is consist of one positioned superior and another positioned anteroinferior. For the dual plating fixation, the plate was positioned on the superior surface of the clavicle according to recommended surgical guidelines (Fig. 1c). The superior plating and anteroinferior plating were positioned as recommended by the manufacturers as demonstrated in Fig. 1a and b. After being positioned as recommended by the manufacturers, the models were put into the ANSYS software for re-meshing, and a four-node tetrahedral three-dimensional element in this study was utilized in the selection of the unit type for the better appropriateness of geometric nonlinear analysis. The numbers of nodes and elements of clavicle and implants are shown in Table 1. And the mechanical properties of clavicle and implants were adopted from previous published reports [17, 19–21] (Table 2).

**Table 1:** Numbers of nodes and elements of bone and implants

Model	Bone	Superior	Anteroinferior	Dual
Node	3356	4253	4275	4923
Element	13124	15673	15719	17458

**Table 2:** Material properties used in finite element models

Materials	Young’s modulus (MPa)	Poisson’s ratio
Cortical bone	17000	0.3
Spongious bone	1000	0.3
Titanium alloy	186400	0.3

### Loading and boundary conditions

**Fig. 2:**
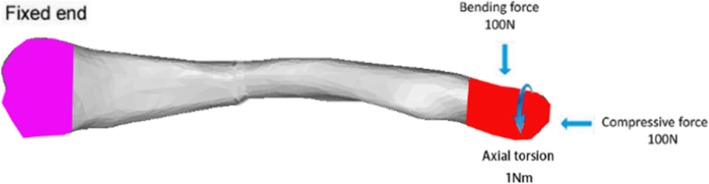
Boundary and loading conditions

Based on the biomechanical behavior of the clavicle, 3 loading modes were simulated in this study [17, 20]. 100 N of cantilever bending, 100 N of axial compression, and 1 Nm of clockwise axial torsion were respectively applied at the lateral end of the clavicle as illustrated in Fig. 2. The sternal end of the clavicle was fixed in all degrees of freedom.

### Analysis

In this study, the computational analysis was done using a commercial finite element software (ANSYS WORKBENCH, ANSYS. Software Corporation, Canonsburg, USA) with the equivalent von Mises stress (EVMS), displacement of the model and implants which was used as the output measures. For statistical analysis, the mean values of stress and displacement between the three models were compared using Student’s *t* test. A *P* < 0.01 was regarded as statistically significant difference.

## Results

### Model validation

**Fig. 3:**
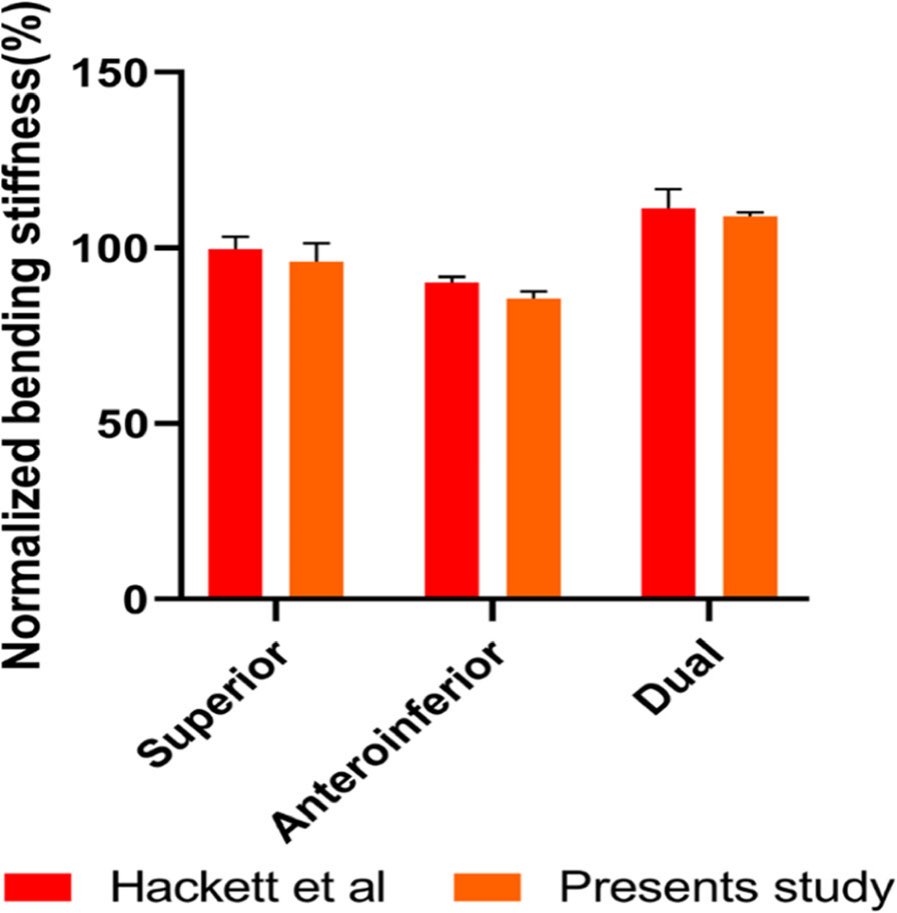
Construct rigidity of three fixation under bending condition compared with the published experimental data. The values obtained for the intact clavicle were set to 100% and served as a reference

The stress distributions in three plates were analyzed and compared with those in model intact clavicle. The results of bending stiffness in our FE model were agreeable with the existing findings. Both results showed similar trends, but with less disparities among different constructs of the FE models (Fig. 3). And this may be attributable to variation in clavicle anatomy and different plate sizes.

### Stress distribution and maximal stress point

**Fig. 4:**
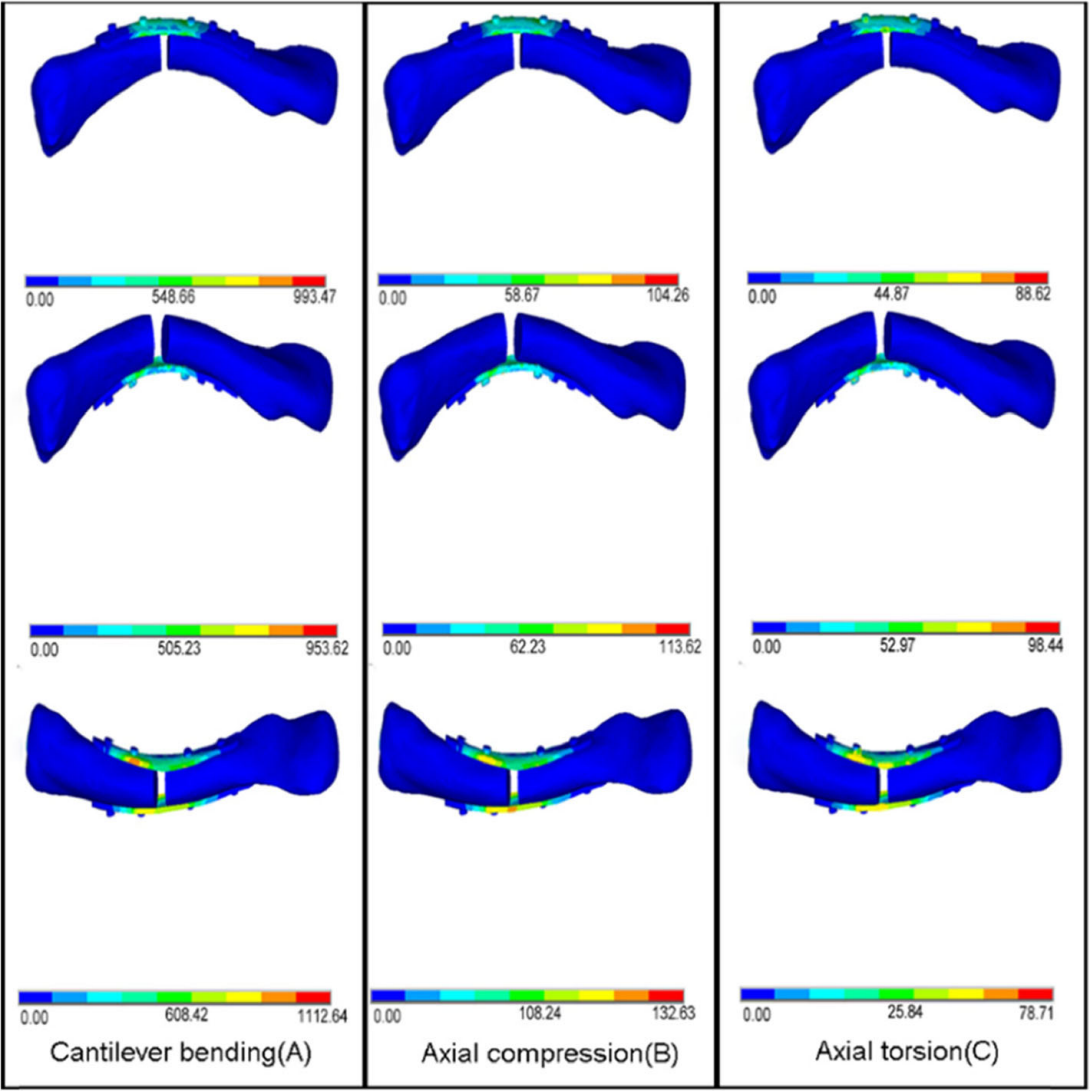
Von Mises stress distribution in the bone of the 3 FE models under 3 loading conditions. Cantilever bending (**a**), axial compression (**b**), and axial torsion (**c**)

The von Mises stress distributions of the intact and fracture models are shown in Table 3. In all loading modes, the three reconstructions led to higher stresses in bone than intact clavicle. Under 100 N of cantilever bending load, the average peak bending stress on the clavicle are showed in Fig. 4a. The maximal stress points are all around the fracture sites. The maximal stress point in the dual plate was 1112.64 MPa; however, the maximal stress point in the superior and anteroinferior plate were 993.47 Mpa and 953.62 Mpa, respectively. And under 100 N of axial compression load on the clavicle. The stress of the dual plate was 132.63 MPa, higher than those of superior plate (104.26Mpa) and anteroinferior plate (113.62Mpa), respectively. (Fig. 4b). With 1 Nm clockwise axial torsion load. The maximal stress from this axial torsion load in the dual plate was 78.71 MPa; however, in the superior plate and anteroinferior plate, the maximal stress was 88.62Mpa and 98.44Mpa, respectively (Fig. 4c). And all maximal stress points are around the fracture sites.

**Table 3:** Peak von Mises stresses of the intact model and three fixations

Model	Implant stress(Mpa)			Bone stress(Mpa)		
	Cantilever bending	Axial compression	Axial torsion	Cantilever bending	Axial compression	Axial torsion
Intact				64.78	11.24	39.44
Superior	993.47	104.26	88.62	79.78	28.34	79.07
Anteroinferior	953.62	113.62	98.44	82.64	30.21	78.63
Dual	1112.64	132.63	78.71	73.24	22.48	54.52

### Structural stiffness

**Fig. 5:**
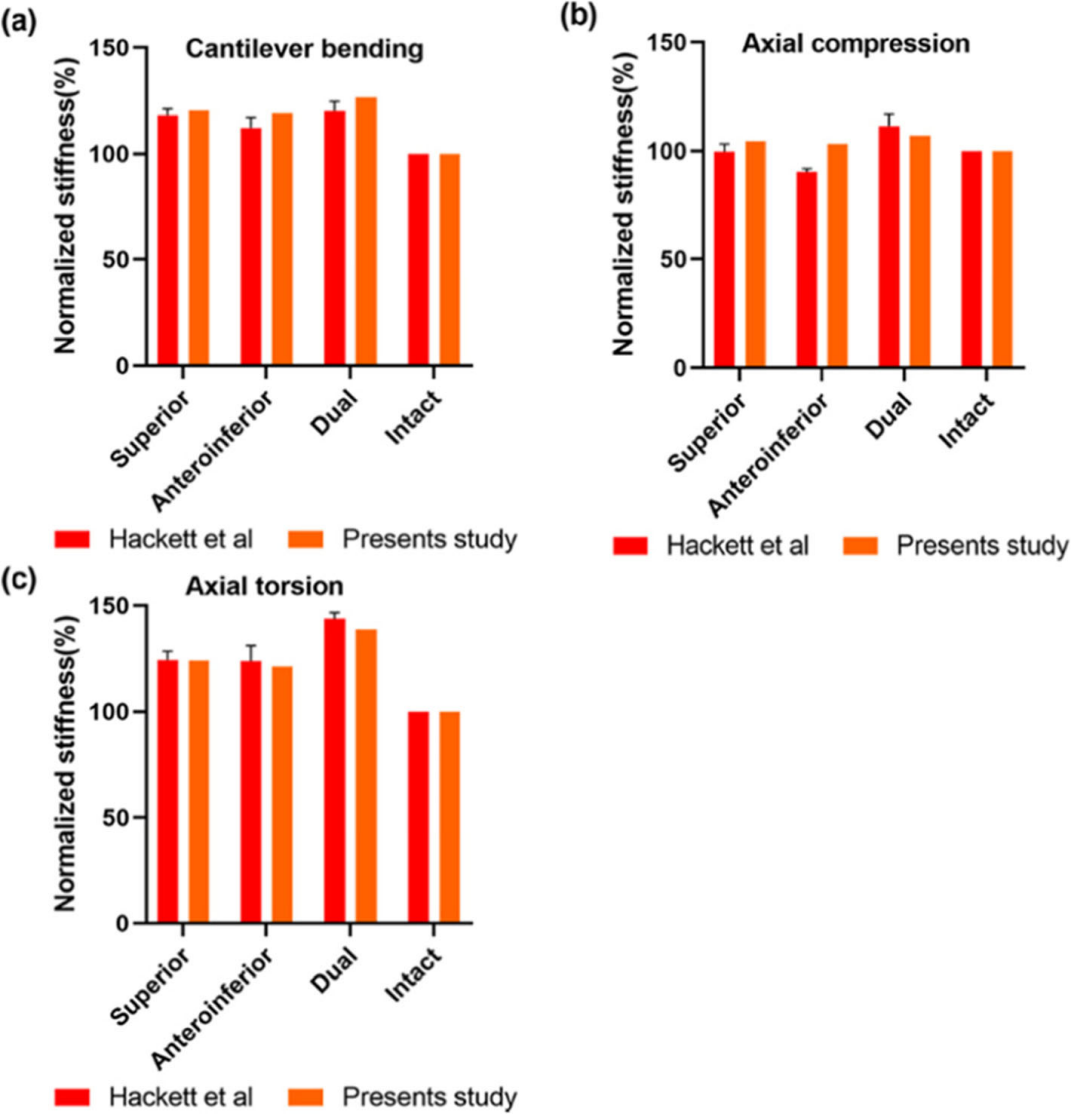
Normalized stiffness of three fixation of the superior (**a**), anteroinferior (**b**), and dual plate (**c**) in 3 loading cases. The values obtained for the spiral plate in axial compression were set to 100% and served as a reference

Figure 5 shows the normalized structural stiffness of different constructs. For the intact clavicle, the bending stiffness was about 25% lower than that of the plate construct. We found that the dual plate model has greater stiffness under cantilever bending loading modes (+ 126.42%), axial compressive mode (106.68%), and axial torsion mode (138.63%). By contrast, the superior plate yielded values of 4.62%, 20.19%, and 23.88% under axial compressive, cantilever bending modes, and axial torsion mode, respectively. The anteroinferior plate yielded values of 3.18%, 18.91%, and 21.13% under axial compressive, cantilever bending, and axial torsion modes, respectively. The results indicated that the structural stiffness of the superior plate and anteroinferior plate was lower than that of the dual plate, and very close to that of the intact clavicle. And the dual plate was a stable fixation for the mid-shaft clavicle fractures.

### Micro-motions

The average displacements for the clavicle fracture are shown in Table 4. The average displacements showed greater similarity of three fixations to the intact clavicle model. However, the dual plate fixation model indicated greater stability for fracture treatment.

**Table 4:** Average displacements of uniform position of each model under axial compressive and cantilever bending loading modes (millimeter)

Model	Intact	Superior	Anteroinferior	Dual
Axial compressive	0.061	0.106	0.089	0.072
Cantilever bending	3.078	0.335	0.389	0.312

## Discussion

Clavicle fractures are relatively common fractures, and over 80% of clavicle fractures involve the midshaft. Clavicle fractures are prevalent in athletes, with several high-profile cases from professional cycling and the National Football League (NFL). With the advancement of open reduction internal fixation techniques, the treatment of midshaft clavicular fractures has become a focus area of contemporary orthopedic research. At present, the plate fixation is the most established method for treatment of midshaft clavicular fractures, including superior plating, anteroinferior plating, and, more recently, dual small plate fixation has caused clinical interest in dual small plating, to minimize hardware irritation and reoperation rates [10, 11, 13, 14, 16]. It is meaningful for surgeons to evaluate the biomechanical performance of implants for the reason of improving the treatment result of clavicle fracture.

However, limited biomechanical data exist, mainly because of difficulties in directly measuring structural complexity, such as the complex attachment of multiple muscles and ligaments and the S-shape of the clavicle itself. In current research, finite element (FE) analysis has been used for the purpose of predicting the influence of specific factors in a given system, with a view to achieving a better understanding of geometrical effects [17], because FE models can effectively focus on a single factor, exclude the effects of other variables. As a result, we used the FE analysis software in this study to estimate three different fixations for treating the midshaft clavicle fractures. And we tried to explore the biomechanism distribution of these three methods.

In the viewpoint of biomechanics, the structures dual small plate fixation is biomechanically similar to superior and anteroinferior single plate fixation with larger clavicle plates. Little differences were noted in cantilever bending, axial compression, and axial torsion between dual plate and either superior or anteroinferior single plate. For construct stability, the dual small plate fixation exhibited the highest stiffness and the least micro-motion. The dual plate model have greater stiffness under cantilever bending loading modes (+ 126.42%) and axial compressive mode (106.68%). By contrast, the superior plate yielded values of 4.62% and 20.19% under axial compressive and cantilever bending modes, respectively. The anteroinferior plate yielded values of 3.18% and 18.91% under axial compressive and cantilever bending modes, respectively. These findings were similar to that of Thomas et al. [16], who found that dual plate fixation was biomechanically similar to superior and anteroinferior single plate fixation.

The distribution of the stress on models was counted through equivalent Von mises stress (EVMS). The concentration of stress found on the superior and anteroinferior single plate was located on the intersection area between the second and third proximal screw which was near the fracture gap, manifesting this screw shared an important contribution for the load transmitted from the cantilever bending, axial compression, and axial torsion. In contrast, on model of the dual plate, the force distribution was more equal than that on the other models. This can be explained by the fact that the dual plate fixation had a bigger cross-sectional area, so the bilateral plate provided a more stable support than superior and anteroinferior single plate fixation which can endure the early weight bearing. What deserves to be mentioned is the stress concentration was found on the cortical regions surrounding the screws. It can be explained by the anti-sliding effect of the screws. In spite of dual small plate may provide better stability, the injury of the periosteum and large incision of the surgery may cause the delayed unions especially in elderly people.

On the other hand, plate prominence and hardware irritation are normally reported as reasons for revision surgeries [3, 13]. However, higher patient cosmetic acceptability has been reported with small plate fixation compared with larger, more prominent plates (95% vs 50%, respectively) [12]. The biomechanical eligibility of dual plating demonstrates that dual plate fixation is a viable option in active patients, including athletes. Therefore, the dual plate fixation probably is a suitable method for young patients whose bones are biomechanically sturdy and has the requirement of early weight bearing.

The limitations of this study included the ideal bonded construct that uses only one standard clavicle model fixing and applying only the constant unidirectional force for all loading conditions. Although these simplifications were helpful for comparing the dual small plate, superior, and anteroinferior single plate fixation, we did not analyze some errors, such as clavicle morphology, in the absence of muscles and ligaments [21].. However, regarding the avoidance of these limitations, we believe our results would be valuable for surgeons to evaluate the biomechanical performance of implants since it can help to improve the treatment result of displaced clavicle fracture. The findings will need to be corroborated by the results of randomized controlled trials including long-term follow-up.

## Conclusion

No significant differences were found between dual plating, superior or anteroinferior single plating in cantilever bending, axial compression, and axial torsion. Dual plating with a smaller plate-screw construct is biomechanically eligible to compare with superior and anteroinferior single plate fixation using larger plate-screw constructs. Dual plating may provide a suitable method for midshaft clavicle fractures and, thus, dual small plate fixation may be preferred for patients requiring an early return to activity.

## Supplementary information

**Additional file 1.** Supplementary fig

## Abbreviations

DICOM: Digital imaging and communications in medicine
FEA: Finite element analysis
