# Supplementary material for: Finite element analysis of dual small plate fixation and single plate fixation for treatment of midshaft clavicle fractures
Source: J Orthop Surg Res. 2020 Apr 15;15:148. doi: 10.1186/s13018-020-01666-x (PMC7161212; doi:10.1186/s13018-020-01666-x)
Supplement: Supplementary file 1 [file 13018_2020_1666_MOESM1_ESM.docx]

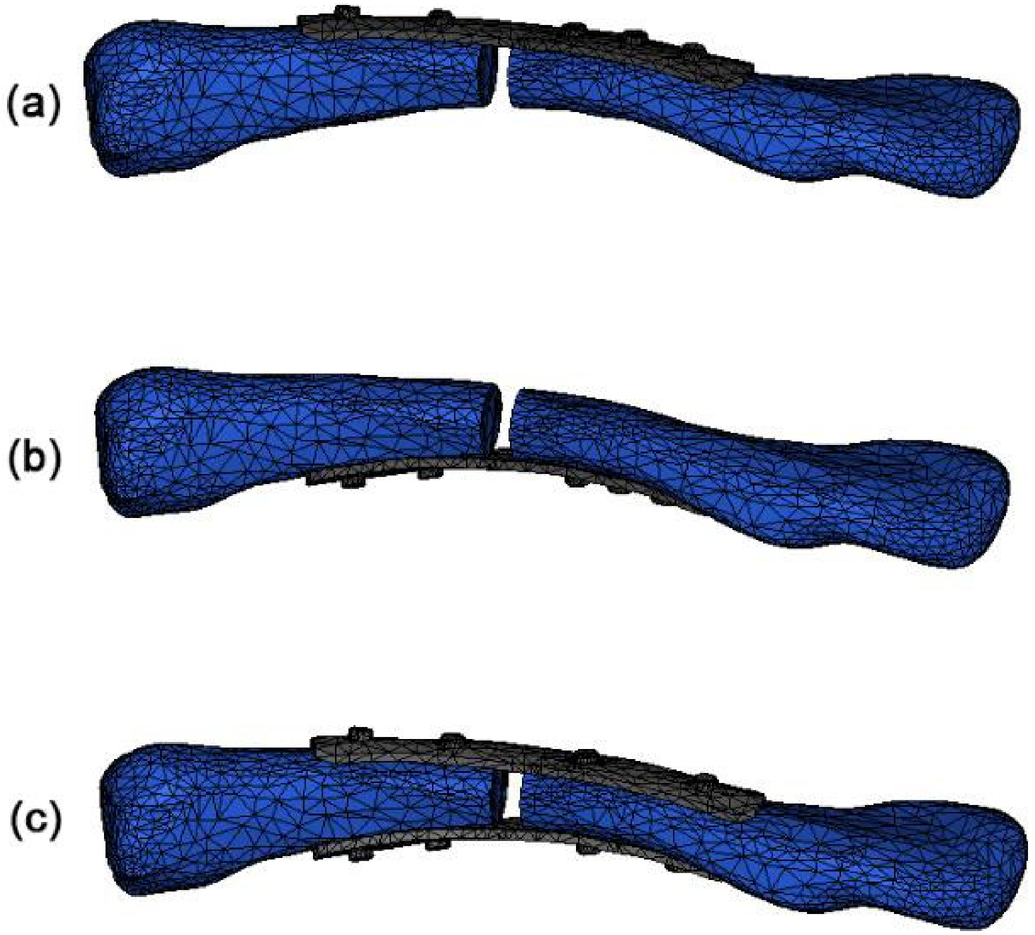
(a)
(b)
(c)
